# A Systematic Review of Cost-Effectiveness Studies on Pancreatic Cancer Screening

**DOI:** 10.3390/curroncol32040225

**Published:** 2025-04-11

**Authors:** Diedron Lewis, Laura Jiménez, Kelvin K. Chan, Susan Horton, William W. L. Wong

**Affiliations:** 1 School of Pharmacy, University of Waterloo, Waterloo, ON N2G 1C5, Canada; william.wl.wong@uwaterloo.ca; 2 Department of Community Health and Epidemiology, Dalhousie University, Halifax, NS B3H 4R2, Canada; laura.jimenez@dal.ca; 3 Odette Cancer Centre, Sunnybrook Health Sciences Centre, Toronto, ON M4N 3M5, Canada; kelvin.chan@sunnybrook.ca; 4 School of Public Health Sciences, University of Waterloo, Waterloo, ON N2L 3G5, Canada; sehorton@uwaterloo.ca

**Keywords:** systematic review, pancreatic cancer screening, cost-effectiveness

## Abstract

Background: Pancreatic cancer (PC) is among the deadliest types of cancer globally. While early detection helps avert adverse outcomes, screening is only recommended for individuals at high risk, specifically those with familial and/or genetic predispositions. The objectives of this study are to systematically review primary studies on the cost-effectiveness of PC screening and to identify the critical factors that influence cost-effectiveness. Methods: This systematic review was performed using PRISMA guidelines. Economic evaluation studies on PC screening were identified from searches on the SCOPUS and PubMed databases. The quality of reporting of the selected articles was assessed according to CHEERS 2022. Using predefined inclusion and exclusion criteria, two reviewers conducted the title–abstract review, full-text review, and data extraction to select relevant articles. The authors’ consensus was used to settle disagreements. The primary outcome was the incremental cost-effectiveness ratio, measured by cost per quality-adjusted life year and cost per life year saved. Results: Nine studies were selected for the final review. Most studies demonstrated that one-time screening for PC among high-risk individuals was cost-effective compared with no screening, while others found annual screening to also be cost-effective. High-risk was generally defined as having a >5% lifetime risk of PC and included individuals with either familial pancreatic cancer (FPC) or genetic susceptibility syndromes such as Peutz–Jeghers Syndrome, hereditary pancreatitis, hereditary non-polypoid colorectal cancer syndrome, familial adenomatous polyposis, and BRCA2 mutations. Individuals with new-onset diabetes (NOD) were also considered high-risk. Screening using mainly endoscopic ultrasound was cost-effective among FPC individuals and those with genetic syndromes. Risk-based screening was also cost-effective among patients with NOD. Conclusion: Screening for PC is cost-effective among selected high-risk individuals. However, cost-effectiveness depends on epidemiological factors, cost, the diagnostic performance of screening tools, and the overall design of studies.

## 1. Introduction

Pancreatic cancer (PC) was the 12th most common cause of cancer incidence in 2022 with an incidence rate of 6.5 per 100,000 and over 510,990 estimated cases globally [1]. Notwithstanding its relatively low incidence rate, PC was the 6th leading cause of cancer mortality across the world in the same year, with a comparable number of deaths estimated at 467,400 deaths [1]. High mortality is linked to the fact that early-stage disease is often asymptomatic, and most diagnoses are established at an advanced stage of the disease, where less than 20% of patients are eligible for resection, and medical interventions are less likely to be successful [2,3,4]. Moreover, disease prognosis is generally unfavourable, with five-year survival rates at the localized and distant stages of disease being 44% and 3%, respectively [5,6].

While screening has the potential to improve the prognosis with earlier detection of disease in the asymptomatic and precursory stages [7,8,9], it is not recommended for the asymptomatic, average-risk population because of the relatively low incidence of disease, the high cost of screening, and concerns around high false-positive rates that may cause patient distress and lead to unnecessary diagnostic follow-up [10,11]. However, targeting high-risk population groups with a family history or an identifiable genetic predisposition to PC is likely a more feasible option since they account for 10–15% of PC cases [9,10,12]. These groups consist of individuals with a greater than 5% lifetime risk of PC or a 5-fold increased relative risk [9,10,12]. They also include (1) individuals with familial pancreatic cancer (FPC) kindreds (see Canto et al. (2013) [12] for a full definition of FPC), (2) individuals with germline mutations in *ATM*, *BRCA1*, *BRCA2*, *CDKN2A*, *PALB2*, *PRSS1*, *STK11*, and *TP53*, (3) individuals with Lynch syndrome, and (4) individuals with Peutz–Jeghers syndrome (PJS) [9,10,12]. Notwithstanding, there is no consensus across various authorities concerning the age at which screening should begin and end for individuals with each of these conditions [9,12].

Individuals with new-onset diabetes (NOD), i.e., those with a PC diagnosis within three years of being diagnosed with diabetes [13,14], are also considered a high-risk group for PC since approximately 80% of PC patients have diabetes or hyperglycemia [13,14]. PC-associated diabetes is part of a paraneoplastic process and can be considered an early indication of PC, as opposed to classic type 2 diabetes [14,15]. Furthermore, PC cases with a non-familial or non-genetic pathology, as in the case of diabetes-related PC, are considered sporadic and account for 85–90% of the incidence of the disease [16,17,18].

One study using data from the International Cancer of the Pancreas Screening Consortium (CAPS) demonstrated that focusing on individuals at high risk for pancreatic ductal adenocarcinoma (PDAC), which accounts for >90% of the cases of PC [13,14], is effective in detecting 90% of resectable tumours and is associated with a 3-year survival rate of 85% [15]. However, a clinical path involving prophylactic pancreatectomy, which is the only curative approach, may also induce insulin-requiring diabetes mellitus and is associated with less favourable mortality and morbidity outcomes [16,17,18,19].

The most common methods of screening that also possess favourable diagnostic capabilities in terms of sensitivity and specificity include endoscopic ultrasound (EUS), magnetic resonance imaging (MRI), magnetic resonance cholangiopancreatography (MRCP), abdominal ultrasound (AU), computed tomography (CT), and positron emission tomography (PET) [20]. However, annual screening using EUS or MRI/MRCP is recommended for high-risk individuals, especially those with a hereditary predisposition, because these strategies detect smaller cystic lesions and stage I PDAC more accurately than CT, and they do so without using harmful ionizing radiation [12,15,19,21].

More recently, risk-prediction models, like the ones based on The Health Improvement Network (THIN) [22,23] and Enriching New-Onset Diabetes for Pancreatic Cancer (END-PAC) [24], are being established to identify individuals with NOD who are at high risk of sporadic PDAC. Common risk stratifying factors for identifying individuals with NOD resulting from a paraneoplastic process, i.e., PDAC-associated diabetes, are age, weight, change in weight, and blood glucose level [22,24,25]. However, the THIN model incorporates other demographic, behavioural, and clinical variables [22]. Using this model, individuals with a specified minimum risk threshold for PDAC-associated diabetes qualify for definitive diagnostic testing as opposed to the entire population with NOD, including those with classic type 2 diabetes, which would be impractical [20,22,24].

Even with advances in the methods used to screen for PC, consensus on a standard screening protocol is still lacking [10,12]. Policymakers have yet to settle on an optimal screening strategy targeting all high-risk subpopulations [10,12], although screening individuals with hereditary/FPC, NOD, and hereditary pancreatitis (HP) is recommended [11,12,19]. Other concerns around the frequency and start age of screening have not been adequately addressed [12,26]. It is likely that, as a consequence of these matters, consensus on the cost-effectiveness of PC is not well-established in the literature, even with a growing number of economic evaluation studies over the years [25,27,28].

This study aims to systematically review economic evaluation studies on PC screening. It adopts a structured and scientifically accepted methodological approach to synthesizing cost-effectiveness studies, unlike its predecessor [29], which used a non-systematic approach. This study also seeks to identify the critical factors that influence cost-effectiveness. Policymakers may benefit from this review as the synthesis would help better inform PC screening decisions based on evidence on cost, effectiveness, and risk factors.

## 2. Materials and Methods

This study adhered to the guidelines set forth by the Preferred Reporting Items for Systematic Reviews and Meta-Analyses (PRISMA). The research protocol was registered with PROSPERO on 14 October 2023, under registration number CRD42023467167. This protocol proposes to conduct reviews of economic evaluation studies on screening for selected gastrointestinal cancers, including pancreatic cancer.

The literature search was conducted in SCOPUS and PubMed with the assistance of two health sciences librarians. Studies published up to December 2024 were considered. Some key search terms were “pancreatic neoplasms”, “pancreatic cancer”, “adenocarcinoma”, “screening”, “early detection”, “economic evaluation”, and “cost-effectiveness analysis”. See Supplementary Materials File S1 for the complete search strategy. All articles were stored in COVIDENCE. A PRISMA flow chart was generated to summarize how the final list of articles was selected. The following inclusion criteria were adopted to select studies for this systematic review:

1. Studies that evaluated the screening of PC in comparison to no screening.
2. Studies investigating populations classified as at average or above-average risk for PC.
3. Studies that reported quality-adjusted life-years (QALY) or life-years saved (LYS) as patient outcomes.
4. Studies employing decision-analytic modelling to evaluate both the long-term effectiveness and cost-effectiveness of interventions aimed at early detection.
5. Studies that presented the Incremental Cost-Effectiveness Ratio (ICER) or offered sufficient data to calculate ICER.
6. Studies that specified cost per QALY, cost per LYS, or cost per utility gained.
7. Studies that included comprehensive economic evaluations.
8. Studies that were published in the English language.

The following parameters were used to exclude studies from this review:

1. Non-original studies.
2. Studies not published in the English language.
3. Grey literature.
4. Systematic reviews, editorials, letters, abstracts, and studies that do not constitute comprehensive health economic evaluations or focus exclusively on follow-up or treatment strategies.

**Figure 1:**
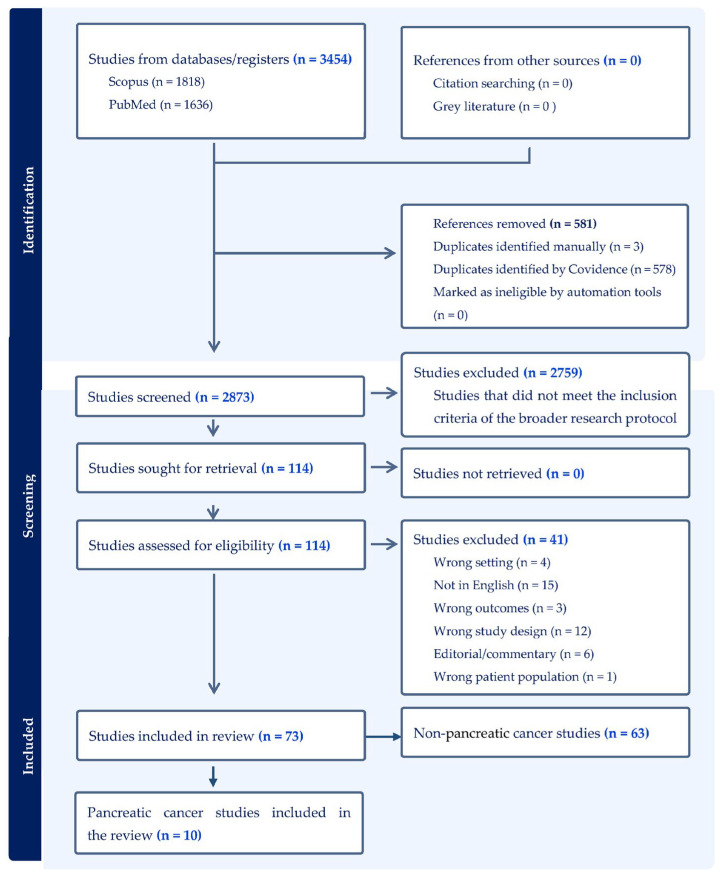
PRISMA diagram of the selection process of the included studies [31].

Using the inclusion and exclusion criteria, two authors (L.J. and D.L.) conducted the title–abstract screening of the studies identified in the literature search. The authors’ consensus was relied upon when disagreements arose. A full-text review was performed on studies selected from the title–abstract screening exercise to identify the final list of studies to include in the systematic review. From the list of selected studies, data on the following variables were extracted: study settings, target populations, study objectives, screening protocols, and details of the economic evaluation model, including the type of model, the perspective of the analysis, the discount rate, the time horizon, the sensitivity analysis conducted, and the willingness to pay (WTP) threshold. Clinical outcomes, screening costs, and study conclusions were also noted. The primary outcome measures of interest were QALYs, LYS, and ICERs. Figure 1 presents the full article-screening process.

The Consolidated Health Economic Evaluation Reporting Standards 2022 (CHEERS 2022) [30] were employed to evaluate the quality of reporting for each study included in the review.

## 3. Results

The search strategy generated 3454 studies, 1818 from Scopus and 1636 from PubMed. A total of 2873 articles were screened after 581 duplicates were removed. Of the 2873 articles, 2759 were excluded because they did not align with the broader research protocol, which focused on reviewing the cost-effectiveness of screening for selected gastrointestinal cancers. Of the remaining 113 studies, 41 were omitted for one of the following reasons: incorrect study setting (*n* = 4), written in a language other than English (*n* = 15), incorrect study outcome (*n* = 3), incorrect study design (*n* = 12), editorial/commentary (*n* = 6), and incorrect patient population (*n* = 1). A further 63 articles were excluded because they focused on the other types of gastrointestinal cancers covered in the research protocol. These include stomach, esophagus, and liver cancers. The remaining ten studies were selected for this systematic review (see Figure 1). Table 1, Table 2 and Table 3 summarize the data extracted from the selected studies.

**Table 1:** Characteristics of included studies.

References	Country	Cancer Risk	Intervention Strategy	Reference Strategy	Study Objective
Wang et al., 2022 [25]	USA	High-risk	One-time screening using 3-year PDAC risk stratification	No screening	To evaluate the cost-effectiveness of this early detection strategy versus the current standard of care for sporadic PDAC among NOD patients and to determine the optimal risk threshold to identify high-risk NOD patients for targeted early detection efforts
Schwartz et al., 2022 [32]	USA	High-risk	One-time risk-based screening using standard-contrast CT	No screening	To develop an early-stage cost-effectiveness model to assess the potential value of risk-based CT pancreatic cancer screening using the END-PAC model in patients with NOD
Rulyak et al., 2003 [27]	USA	High-risk	One-time screening with EUS	No screening	To detect pancreatic dysplasia among members of high-risk familial pancreatic cancer kindreds might be cost-effective
Peters et al., 2024 [33]	USA	High-risk	MRI with EUS referral one-time, every 5 years, every 2 years, and annually	No screening	To evaluate the potential cost-effectiveness of combined MRI and EUS screening for pancreatic PDAC among high-risk populations
Kumar et al., 2021 [26]	USA	High-risk	One-time index EUS	No screening	To evaluate which factors make index EUS a potentially cost-effective strategy in identifying lesions in patients at high risk of developing PDAC, i.e., individuals with at least a 5% lifetime risk of pancreatic cancer
Kowada 2022 [34]	Japan	High-risk	Annual screening using either MicroRNA, CA 19-9, AU, MRI, EUS, CT, or PET	No screening	To assess the cost-effectiveness of microRNA compared with CA 19-9, AU, MRI, EUS, CT, PET, and no screening for pancreatic cancer screening in patients with diabetes
Kowada 2020 [35]	Japan	High-risk	One-time screening using either MRI, EU, CT, or PET, and no screening	One-time screening using AU	To assess the cost-effectiveness of AU, MRI, EUS, CT, PET, and no screening for pancreatic cancer screening in familial high-risk individuals
Joergensen et al., 2016 [36]	Denmark	High-risk	Annual EUS, MRI, MRCP, CT	No screening	To establish a screening program for high-risk groups (i.e., persons with hereditary pancreatitis (HP) or with a predisposition to HP and individuals with first-degree relatives with familial pancreatic cancer) and to evaluate its cost-effectiveness
Draus et al., 2023 [37]	Sweden	Average-risk and high-risk	One-time screening using a hypothetical blood-based biomarker test	Not reported	To evaluate the cost-effectiveness of screening for pancreatic cancer in Sweden using a hypothetical blood-based biomarker test with predetermined sensitivity and specificity in the general population and selected high-risk cohorts
Corral et al., 2019 [28]	USA	High-risk	Annual EUS or MRI	No screening	To perform an economic analysis to identify the different clinical, as well as cost determinants of pancreatic cancer surveillance in high-risk individuals, i.e., individuals with at least 5% lifetime risk of pancreatic cancer

EUS—endoscopic ultrasound; PDAC—pancreatic ductal adenocarcinoma; AU—abdominal ultrasound; CT—computed tomography; MRI—magnetic resonance imaging; PET—positron emission tomography; CA 19-9—carbohydrate antigen 19-19; NOD—new-onset diabetes; END-PAC—Enriching New-Onset Diabetes for Pancreatic Cancer; MRCP—magnetic resonance cholangiopancreatography; PDAC—Pancreatic ductal adenocarcinoma.

**Table 2:** Summary of study methods of included studies.

References	Target Group: Age Range and Risk Condition	Analytical Model	Cycle Length	Time Horizon	Compliance	Perspective	Discount Rate	Source of Clinical Input
Wang et al., 2022 [25]	≥50 years with NOD	Markov	3 months	Lifetime	Not reported	Healthcare sector	3%	Literature, published data, SEER
Schwartz et al., 2022 [32]	≥50 years with NOD	Markov	Monthly	Lifetime	Not reported	Limited US healthcare payer	3%	Literature (Enriching New-Onset Diabetes for Pancreatic Cancer (END-PAC) risk model validation study; Pancreatic Cancer Action Network (PanCAN) trial), SEER
Rulyak et al., 2003 [27]	50-year-olds with FPC	Decision tree	Not applicable	Lifetime	Not reported	Third-party and societal	3%	Literature, Familial Pancreatic Cancer Screening Program database, consensus of two experts
Peters et al., 2024 [33]	≥40 years with germline mutations: *PALB2*, *BRCA1*, Lynch syndrome, *ATM*, *BRCA2*, *TP53*, *CDKN2A*, or *STK11*	Markov mode	1 month	Lifetime	100%	Healthcare sector	3%	SEER, 2019 Medicare and Medicaid data, literature
Kumar et al., 2021 [26]	55-year-olds with FPC	Decision tree	Not applicable	Lifetime	Not reported	Third-party	3%	Literature, Cancer of the Pancreas Screening (CAPS) trial, Centre for Medicare Services, expert opinion
Kowada 2022 [34]	40–70 years with either NOD, LSD, or LSD with IPMN	Markov	1 year	Lifetime	100%	Healthcare payer	3%	Literature, MEDLINE, Japanese statistics
Kowada 2020 [35]	50-year-olds with FPC	Markov	1 year	Lifetime	Not reported	Healthcare sector	3%	Literature, MEDLINE, Japanese statistics
Joergensen et al., 2016 [36]	>30 years with HP and >50 years with FPC patients	Prospective cohort analysis	Not applicable	2006–2014	100%	Not reported (health program intervention)	4%	Danish Diagnose Related Group, study data
Draus et al., 2023 [37]	General population 50–79 years, daily smokers 50–79 years, FPC or hereditary PC 40–79 years, NOD 50–79 years, early symptoms of PC 40–79 years	Not reported	Not reported	Not reported	Not reported	Social economy	3%	Literature, databases
Corral et al., 2019 [28]	40-year-olds with either FPC, genetic syndromes (*BRCA 1* and *2*), PJS, HP, FAMMM, Lynch syndrome, Li Fraumeni, Familial adenomatous polyposis, or NOD	Markov	1 year	Lifetime	Not reported	Third-party payer	3%	Literature, life tables, SEER

FPC—familial pancreatic cancer; HP—hereditary pancreatitis; SEER—Surveillance, Epidemiology, and End Results; LSD—long-standing type 2 diabetes; IPMN—intraductal papillary mucinous neoplasm, PJS—Peutz–Jeghers syndrome, FAMMM—familial atypical mole melanoma; BRCA1—Breast cancer type 1; BRCA2—Breast cancer type 2; PALB2—Partner and localizer of BRCA2; ATM—Ataxia-telangiectasia mutated gene; TP53—Tumor protein 53; CDKN2A—Cyclin-dependent kinase inhibitor 2A; STK11—Serine-threonine kinase 11.

**Table 3:** Summary of outcomes of included studies.

References	Intervention Strategy	Reference Strategy	Year/Currency	Incremental Cost-Effectiveness Ratio	Willingness-to-Pay	Sensitivity Analysis	Sensitive Variables-1-Way SA	Probabilistic Sensitivity Analysis	CHEERS Checklist
Wang et al., 2022 [25]	Screening through 3-year PDAC risk stratification	No screening	2020 USD	Riskthreshold-1%: $116,910.97/QALY, Threshold-2%: $63,045.49/QALY, Threshold-3%: $47,948.20, Threshold-4%: $31,389.02, Threshold-5%: $5407.44,	$100,000/QALY and $150,000/QALY	1-way SA and PSA	The proportion of PDACs detected at local stage, costs of treatment for metastatic PDAC, utilities of local and regional cancers, and sensitivity of screening	1% and 2% risk thresholds CE 30.6% and 20.4% of the times at WTP threshold of $150,000/QALY and 27.3% and 22.8% of the times at WTP threshold of $100,000/QALY	Not reported
Schwartz et al., 2022 [32]	Risk-based screening of patients with NOD using standard-contrast CT	No screening	2020 USD	Screening: $65,076/QALY	$100,000/QALY	1-way SA and PSA	The proportion of screen-detected PAC cases that are resectable, the health state utility for resectable PAC from 6 months after surgery to progression, and the proportion of clinically detected PAC cases with distant-stage disease	Screening CE > 99% of the time	Not reported
Rulyak et al., 2003 [27]	One-time screening with EUS	No screening	2000 USD	Screening: $16,885/LYS	Not reported	1-way SA and 2-way SA	Prevalence of dysplasia, sensitivity of EUS and ERCP, life expectancy (after pancreatectomy)	Not reported	Not reported
Peters et al., 2024 [33]	MRI with EUS referral one-time, every 5 years, every 2 years, and annually	No screening	2019 USD	Men: -*STK11* (RR 28) annual screening from 40 years: $69,000/QALY -*CDKN2A* (RR 12.33) annual screening from 55 years: $82,000/QALY Women: -*STK11* (RR 28) annual screening from 45 years: $45,000/QALY	$100,000/QALY	1-way SA	Specificity of screening, screening cost	Not reported	Not reported
Kumar et al., 2021 [26]	One index EUS	No screening	2018 USD	Screening: $82,669/QALY	$100,000/QALY	1-way SA and 2-way SA	Lifetime risk of PDAC, probability of future PDAC after normal index EUS, and probability of a missed lesion, length of survival after resection	Not reported	Not reported
Kowada 2022 [34]	MicroRNA, CA 19-9, AU, MRI, EUS, CT, and PET	No screening	2020 USD	LSD: AU: $14,968–19,540/QALY, NOD: MicroRNA: $52,611–$68,752/QALY, LSD with IPMN: MicroRNA: $10,130–12,911/QALY	$100,000/QALY	1-way SA, 2-way SA, PSA	Cost of microRNA in patients with LSD, pancreatic cancer incidence in all diabetic patients	AU was 60% to 76% cost-effective in patients with LSD, and microRNA was 42% to 54% cost-effective for NOD and 76% to 78% cost-effective for LSD having IPMN at a WTP threshold of $100,000/QALY	Not reported
Kowada 2020 [35]	MRI, EUS, CT, PET, and no screening	AU	2018 USD	AU is cost-effective (USD 11,035, 17.4875 QALY)	$50,000/QALY	1-way SA, PSA	Incidence of pancreatic cancer	AU is cost-effective 76% of the time at a WTP threshold of $50,000/QALY	Not reported
Joergensen et al., 2016 [36]	Annual EUS or MRI, MRCP or CT, if EUS is not possible	No screening	2015 USD	FPC: $38,785/QALY, HP: $58,647/QALY, Total: $42,128/QALY	$50,000/QALY	Not reported	Risk stratification of the patients offered screening, the performance characteristics of EUS and EUS with FNA or other modalities used as well as the mortality rate after a total pancreatectomy	Not reported	Not reported
Draus et al., 2023 [37]	One-time screening using a hypothetical blood-based biomarker test	Not reported	2018 EUR	Not reported	EUR100,000/QALY	Not reported	Not reported	Not reported	Not reported
Corral et al., 2019 [28]	EUS, MRI	No screening		Annual screening of (1) high-risk individuals: EUS: $13,200/QALY, MRI: dominant strategy; and (2) highest-risk individuals: EUS: dominant strategy, MRI: $7847/QALY	$100,000/QALY	1-way SA, PSA	Age, diagnostic performance of imaging tools	MRI CE > 50% of the time, EUS CE more than 45% of the time	Not reported

QALY—quality-adjusted life year; 1-way SA—one-way sensitivity analysis; PSA—probabilistic sensitivity analysis; CE—cost-effectiveness; WTP—willingness-to-pay; LYS—life-years saved; EUS—endoscopic ultrasound; ERCP—endoscopic retrograde cholangiopancreatography; PDAC—pancreatic ductal adenocarcinoma; AU—abdominal ultrasound; CT—computed tomography; MRI—magnetic resonance imaging; PET—positron emission tomography; CA 19-9—carbohydrate antigen 19-19; LSD—long-standing type 2 diabetes; NOD—new-onset diabetes; IPMN—intraductal papillary mucinous neoplasm; FPC—familial pancreatic cancer; HP—hereditary pancreatitis; MRCP—magnetic resonance cholangiopancreatography; FNA—fine needle aspiration; CDKN2A—Cyclin-dependent kinase inhibitor 2A; STK11—Serine-threonine kinase 11.

### 3.1. Overview of Selected Studies: Target Population and Screening Strategies

The selected studies in this systematic review share the common objective of determining the cost-effectiveness or cost-utility of screening for pancreatic cancer among high-risk groups. The high-risk groups that were of focus include individuals at risk of FPC, individuals with diabetes (i.e., NOD or long-standing type 2 diabetes (LSD)), individuals with a hereditary predisposition, and individuals with early symptoms of pancreatic cancer. Five studies explicitly stated PDAC as the PC subtype of interest [25,26,28,36]. The selected studies were also based on different country populations; six from the USA [25,26,27,28,32], two from Japan [34,35], and one each from Sweden [37] and Denmark [36], respectively (see Table 1).

EUS was modelled as the primary screening strategy among most of the selected studies (*n* = 6). Other screening tools used were AU, CT, MRI, PET, carbohydrate antigen 19 (CA 19-9), microRNA, a blood-based biomarker, and risk-stratified screening tools. In contrast, no screening was the primary reference strategy. There was consensus among the selected studies that PC screening was cost-effective; however, this result must be taken in the context of the screening tool used and the target subgroup among the high-risk population (see Table 1).

The selected studies were grouped into three categories based on pancreatic cancer risk factors. These categories are (1) studies that focused on screening patients with diabetes (*n* = 3), (2) studies that focused on screening patients with FPC (*n* = 3), and (3) studies that focused on multiple risk factors (*n* = 4).

### 3.2. Cost-Effectiveness of Studies Focusing on Screening Patients with Diabetes

For the studies that focused on screening patients with diabetes, the emphasis was particularly on those with NOD [25,32,34]. Two of the three studies in this category proposed screening strategies based on risk threshold models [25,32].

Wang et al. (2022) used a risk prediction model along with data from the THIN cohort [23] to identify individuals with NOD over 50 years old in the USA who were at high risk of PDAC [25]. This one-time intervention strategy stratified cohort members into high- and low-risk groups based on risk thresholds ranging from 0.5% to 5% [25], where risk level was determined by the following factors: age, body mass index, change in body mass index, smoking, use of proton pump inhibitors, and anti-diabetic medications, as well as levels of hemoglobin A1c, cholesterol, hemoglobin, creatinine, and alkaline phosphatase [23]. This study also found that at a WTP threshold of $100,000/QALY, only risk thresholds between 2% and 5% were cost-effective from a healthcare sector perspective (ICER: $5407.44–63,045.49/QALY (2020 USD)) [25]. In fact, at this lower WTP threshold, 1% and 2% risk thresholds were cost-effective only 27.3% and 22.8% of the time, respectively (see Table 1, Table 2 and Table 3).

In a similar study, Schwartz et al. (2022) used the END-PAC risk model [24] to stratify NOD patients who were at least 50 years old based on the following factors to determine the risk of pancreatic cancer: change in weight, change in blood glucose, and the age at the onset of diabetes. Assuming a one-time application of the risk model, individuals with an END-PAC score greater than zero would undergo CT screening [32]. This study demonstrated that screening those with a positive END-PAC score was cost-effective > 99% of the time from a limited USA healthcare payer perspective, with an ICER of $65,076/QALY at a WTP threshold of $100,000/QALY (2020 USD) [32] (see Table 1, Table 2 and Table 3).

Meanwhile, in Kowada (2022), the target population included Japanese patients with different diabetic conditions, namely, NOD, LSD (i.e., diabetes for more than five years), and LSD with intraductal papillary mucinous neoplasm (LSD-IPMN) [34]. This study demonstrated that for persons aged 40, 50, 60, and 70 years, annual AU was cost-effective for those with LSD (ICER: $14,968–19,540/QALY), while annual microRNA was cost-effective for those with NOD and those with LSD-IPMN (ICERs: $52,611–$68,752/QALY and $10,130–12,911/QALY, respectively) at a WTP threshold of $100,000/QALY (2020 USD) [34]. Cost-effectiveness was estimated from a healthcare payer perspective [34] (see Table 1, Table 2 and Table 3).

### 3.3. Cost-Effectiveness of Studies Focusing on Screening Individuals with FPC

Although three studies focused on screening individuals with FPC, they each had varying definitions of FPC; yet, they all demonstrated that screening was cost-effective [26,27,35]. Rulyak et al. (2003) adopted the definition proposed in an earlier study (Brentnall 2000) that defined FPC as either:

1. An individual who has two or more first-degree relatives (FDRs) with PC.
2. An individual who has one first-degree relative (FDR) diagnosed with PC at an early age (≤50).
3. An individual who has two or more second-degree relatives with PC, one of whom developed it at an early age [38].

Rulyak et al. (2003) estimated that, in the USA, one-time screening of 50-year-old individuals with FPC using EUS was (1) cost-effective from a third-party-payer perspective (ICER: $16,885/LYS (2000 USD) and (2) a dominant strategy from a societal perspective [27]. A WTP level was not reported in this study (see Table 1, Table 2 and Table 3).

In Kumar et al. (2021), an individual with FPC was described as having kindred with ≥2 FDRs with PDAC, with an increasing risk of PC as the number of affected relatives increases, and a baseline lifetime risk of 5% [26]. This study also revealed that one-time EUS at the age of 55 years was cost-effective among individuals with FPC from a USA third-party perspective compared with no screening (ICER: $82,669/QALY (2018 USD) at a WTP threshold of $100,000/QALY) [26] (see Table 1, Table 2 and Table 3).

Meanwhile, Kowada (2020) defined FPC individuals as persons with a high risk of pancreatic cancer originating from families with ≥3 family members with PC, with at least two being FDRs [35]. In this study, one-time AU screening among 50-year-old individuals with FPC was cost-effective 76% of the time at a WTP level of $50,000/QALY (2018 USD) from a Japanese healthcare sector perspective. Comparator strategies were not cost-effective, including CT, MRI, PET, EUS, and no screening [35]. However, the one-way sensitivity analysis revealed that EUS was cost-effective if the incidence of PC was between 0.008 and 0.016 within high-risk populations with a familial predisposition [35] (see Table 1, Table 2 and Table 3).

### 3.4. Cost-Effectiveness of Studies Focusing on Multiple Risk Factors

Four studies also focused on multiple risk factors [28,33,36,37]. While they all demonstrated that screening was cost-effective, they targeted different population groups and utilized different screening methods.

Joergensen et al. (2016), for example, using data from a national screening program in Denmark between 2006 and 2014, demonstrated that annual screening using EUS (or MRI/MRCP or CT if EUS was not feasible) was cost-effective for a selected group of 71 patients (≥50 years), consisting of 31 patients with HP or a predisposition to HP (≥30 years), and 40 FDRs of patients with FPC [36]. The ICER for both groups combined was estimated to be $42,128/QALY (2015 USD) at a WTP threshold of $50,000/QALY. However, when disaggregated, only screening the FPC group was cost-effective (ICER: $38,785/QALY) [36] (see Table 1, Table 2 and Table 3).

In the Draus et al. (2023) study, the cost-effectiveness of a hypothetical blood-based biomarker screening test was evaluated from a Swedish social economy perspective across five population groups: the general population aged 50–79 years, daily cigarette smokers aged 50–79 years, individuals at risk for hereditary and/or familial pancreatic cancer aged 40–79 years, individuals with NOD aged 50–79 years, and individuals with early symptoms of pancreatic cancer aged 40–79 years [37]. The results of this study showed that should the sensitivity and specificity levels of the hypothetical test be 80% to 95%, one-time screening of population groups with NOD, hereditary, and/or familial pancreatic cancer and early symptoms of pancreatic cancer would be cost-effective, falling within the €100,000/QALY WTP threshold (2018 €) [37] (see Table 1, Table 2 and Table 3).

Meanwhile, Corral et al. (2019) adopted the CAPS definition of high-risk individuals, which consists of individuals with >5% lifetime risk of pancreatic cancer, including persons with FPC, PJS, HP, Lynch syndrome, and selected gene mutations [12,28]. In the Corral et al. study, annual EUS and annual MRI, starting at the age of 40 years, were illustrated to be cost-effective given a WTP threshold of $100,000/QALY (2017 USD) from a USA third-party-payer perspective [28]. However, for cases with a relative risk from 5% to 20% (defined as high-risk), MRI was the dominant strategy (cost: $27,617, QALY: 21.5), although EUS was cost-effective with ICER: $13,200/QALY. These cases include individuals with FPC with one or two FDRs with PC, hereditary non-polypoid colorectal cancer syndrome, familial adenomatous polyposis, and BRCA2 mutations [28]. Meanwhile, for cases with a relative risk greater than 20% (defined as the highest risk), EUS was the dominant strategy, although MRI was also cost-effective (ICER: $7847). Cases in this group include those with FPC and ≥3 FDRs with PC, as well as individuals with HP and those with PJS [28] (see Table 1, Table 2 and Table 3).

Peters et al. (2024), in a similar study that focused on screening individuals with selected germline mutations, found that annual screening using MRI (and EUS follow-up for cases of positive indication) was cost-effective for men and women with Serine-threonine kinase 11 and a PDAC relative risk of 28 (ICER: $69,000/QALY for men starting from the age of 40 and $45,000/QALY for women starting from the age of 45 (2019 USD), respectively) [33]. Screening starting from the age of 55 was also cost-effective for men with Cyclin-dependent kinase inhibitor 2A and a PDAC relative risk of 12.33 (ICER: $82,000/QALY). Cost-effectiveness was measured from a healthcare sector perspective (see Table 1, Table 2 and Table 3).

### 3.5. Study Type, Models, Input Parameters, and Sensitivity Analysis

The selected studies adopted different methodological approaches to perform their various economic evaluations, namely, state transition Markov models [25,28,32,33,34,35], decision tree models [26,27], and a prospective cohort design (described earlier) [36]. Further differences can be identified in several of the variables used in the models across each study, including health states, patient populations, cycle lengths, and time horizons (see Table 2).

Of the six studies that presented state transition models [25,28,32,33,34,35], three focused on patients with diabetes [25,32,34]; with two specifically on patients with NOD [25,32]. These three studies also modelled different health states for diabetic patients. For example, Kowada (2022) disaggregated cancer stages according to the TNM Staging/Overall Stage Grouping system, identifying stages I to IV of the disease [34]. Meanwhile, Wang et al. (2022) used the Surveillance, Epidemiology, and End-Results (SEER) General Summary Staging System, which distinguishes local, regional, distant, and unknown stages of the disease [25]. Schwartz et al. (2022) described the main health states as resectable and unresectable PC [32]. In contrast, Corral et al. (2019) focused on multiple high-risk factors and therefore the stages of PC were not disaggregated, but the model distinguished between low- and high-risk lesions [28].

Most model-based studies also modelled follow-up procedures following a positive test result from screening. This included conducting confirmatory diagnostic tests, such as EUS fine needle aspiration biopsy [28,36] and endoscopic retrograde cholangiopancreatography. Follow-up CT [35] and MRI [34,35] were modelled in selected Japanese-based studies. Surgical intervention, including total pancreatectomy, was also captured in most studies following a positive confirmatory test result, where the indication was eligible for resection [27,28,34,35,36].

Annual cycle lengths were used primarily to represent the time individuals can transition between health states. However, Schwartz et al. (2022) [32] and Peters et al. (2024) [33] used cycle lengths of one month while Wang et al. (2022) [25] used a three-month cycle length (see Table 2).

The studies that developed decision trees presented the options and outcomes along the paths of PC screening compared to no screening. One study focused on high-risk individuals in general [26], and the other on individuals at risk of FPC [27] (see Table 2).

Screening typically started at the age of 50 years in most studies [25,27,32,35,36,37]. Other start ages, such as 30, 40, and 55, were also used [26,28,33,34,36,37]. Most of the studies adopted a lifetime horizon, except for Joergensen et al. (2016) [36], which followed a prospective cohort, and Corral et al. (2019) [28], which did not specify a horizon. The most common discount rate was 3%, except for Joergensen et al. (2016), who used 4%. Across all studies, input parameters were derived primarily from published literature, various databases, and randomized controlled trials (see Table 2).

In the univariate sensitivity analyses, some of the more sensitive variables that impacted cost-effectiveness were the proportion of PDACs detected at the local stage and the distant stage, the cost of treating metastatic PDAC, the utilities of local and regional cancers, the prevalence of dysplasia, life expectancy (after pancreatectomy), the lifetime risk of PDAC, the incidence of pancreatic cancer (especially in diabetic patients), the age of patients, and the diagnostic performance of screening tools (i.e., the levels of sensitivity and specificity).

Cost-effectiveness was also established on favourable levels of sensitivity and specificity of each respective screening tool. For example, for EUS, the lowest sensitivity and specificity levels reported were 71.25% [26] and 86% [34,35], respectively. Another study demonstrated that screening tests with sensitivity and specificity levels ranging between 80% and 95% are more likely to be cost-effective [37] (see Supplementary Materials Table S1).

### 3.6. Results on Quality of Reporting

Online Resource 2 presents the CHEERS 2022 checklist on the quality of reporting of the selected studies of this systematic review conducted by the authors [30]. While the selected studies reported on most of the relevant items in the checklists, two items were consistently not reported: items 21 and 25. These items address engagements with patients and other stakeholders in conducting studies and are not necessarily relevant for model-based studies where cohorts are typically hypothetical. Nevertheless, none of the studies reported their own CHEERS checklist. All studies reported conflicts of interest and sources of funding (see Table 3 and Supplementary Materials Table S2).

## 4. Discussion

This systematic review synthesized the relevant studies on the cost-effectiveness of PC screening. The findings provided circumstances under which PC screening may be cost-effective. Several key factors proved to be most influential in determining cost-effectiveness. These included critical variables such as screening methods, the selected high-risk population groups, the country being investigated, disease detection rates, study perspective, treatment costs, the sensitivity and specificity of screening tools, health utilities, disease prevalence and incidence, the risk of PDAC, the WTP threshold, and patient age. Nonetheless, there was consensus among the selected studies that PC screening was cost-effective compared with no screening, and this was demonstrated from multiple perspectives.

Other key insights can be gleaned from the outcomes of the systematic review. Firstly, EUS was demonstrated to be a cost-effective screening tool for individuals with FPC, with three studies modelling one-time screening [26,27,35] and two studies modelling annual screening [28,36]. EUS was also a dominant strategy for screening patients with HP [28,36] and PJS [28], indicating the test’s versatility.

Secondly, for diabetic patients, particularly those with NOD, one-time risk-based screening was cost-effective [25,32]. Alternatively, annual microRNA screening was also cost-effective for NOD patients [34]. However, annual AU was preferred for patients with LSD [34]. Anthropometric variables such as age and weight form the basis of these risk-based models [25,32], but accounting for other relevant factors, including behavioural and lifestyle characteristics, medication use, and laboratory outcomes of hemoglobin A1C, hemoglobin, total cholesterol, creatinine, and alkaline phosphatase, can potentially better reflect the overall risk of NOD-associated PC [22].

Thirdly, quantifying risk levels is potentially a critical factor in determining cost-effectiveness. While screening is recommended for high-risk individuals, i.e., those with > 5% lifetime risk of PC, Corral et al. (2019) demonstrated that cost-effectiveness changes when this population is further disaggregated into high-risk individuals (those with 5–20% lifetime risk) and highest-risk individuals (i.e., those with >20% lifetime risk). For example, MRI was a dominant strategy for individuals in the former group, which included those with FPC with 1–2 FDRs, hereditary non-polypoid colorectal cancer syndrome, familial adenomatous polyposis, and BRCA2 mutations [28]. Meanwhile, EUS was a dominant strategy for individuals in the latter group, which included those with FPC and ≥ 3 FDRs with PC, as well as individuals with HP and those with PJS [28].

Fourthly, there was no consensus on the age at which screening should commence. Although most studies adopted the age of 50 years [25,27,32,35,36,37], others used 30, 40, and 55 years. Clinicians should, therefore, follow the recommendations of their respective authorities on this matter.

The results of the systematic review are generally consistent with current screening recommendations that propose annual screening using EUS or MRI/MRCP for FDR of patients with PC from familial PC kindred with at least two affected FDRs, patients with PJS, and p16, BRCA2 and hereditary non-polyposis colorectal cancer mutation carriers with ≥1 affected FDR [12,15,19,21].

The results of this review were also comparable with those provided by Wang et al. (2024), which was a non-systematic review of the cost-effectiveness of surveillance for hereditary pancreatic cancer [29]. This study concluded that both EUS and MRI are potentially cost-effective strategies compared with no surveillance, particularly among individuals in selected pathogenic germline variant/familial groups with a >10% risk of PC [29]. Unlike the Wang et al. study (2024), this present study adopted a more rigorous and widely accepted methodological approach for conducting a review of the literature on the cost-effectiveness of PC screening. In addition to the common variables investigated, like the country setting, population, follow-up period, screening strategy, ICER, and WTP, this present study examined other reported variables, such as screening start age, analytical modelling, cycle length, study perspective, discount rate, sensitivity analysis, and the CHEERS checklist.

While these results are instructive for researchers and policymakers, it is important to distinguish between screening and surveillance efforts. CAPS explains that screening is aimed at detecting and treating stage I PDAC/T1-2N0M0 along with related high-grade dysplastic precursor lesions: pancreatic intraepithelial neoplasia and intraductal papillary mucinous neoplasm [12,21]. For individuals with no concerning indications, screening should be repeated annually once patients are candidates for resection [12,19,21]. However, this recommendation only applies under academic conditions and is to be carried out by an experienced multidisciplinary team [19,21]. Meanwhile, surveillance is recommended for concerning abnormalities that do not appear to be malignant, with repeated image testing every three or six months depending on the size, number, type, and growth rate of lesions [9,19,21]. Economic evaluation models that account for treatment and resection of early-stage cancer and surveillance of high-risk individuals, such as those with FPC, germline mutations, Lynch syndrome, NOD, and PJS, would capture a more comprehensive care pathway in evaluating cost-effectiveness.

Even in recognizing the many subgroups at high risk of PC, studies have primarily focused on those with FPC and NOD [21,39]. It is estimated that 10–15% of the cases of PDAC are linked to hereditary risk factors, including 5–10% of cases having a familial history [21,40,41]. Also, approximately 80% of patients in the asymptomatic stage of PC have either hyperglycemia or diabetes [39]. Expanding future research to capture other high-risk groups where the lifetime risk of PC is also > 5% (as defined by screening recommendations) [12,19,21] would also be informative. Special attention can also be given to the approximately 10% of PC cases that are not of the PDAC subtype.

In addition to being cognizant of etiological factors, interventions should also consider the distribution of the incidence of PC worldwide. Although the incidence of the disease is generally low globally (<7 cases per 100,000 persons) [1], and likely even lower in settings where screening is not conducted, the incidence rates were highest in Europe and North America [1]. Japan and Finland recorded the highest incidence rates in 2022, with 37.92 and 26.95 cases per 100,000 persons, respectively. [1]. Jurisdictions with higher rates may benefit most from secondary intervention programs. Economic evaluation studies in these locations would also build evidence and consensus on the cost-effectiveness of screening and surveillance.

This study has several limitations. Articles not published in the English language were excluded from the review, thereby restricting the pool of studies that could potentially be included. Similarly, the literature search was executed in only two databases, limiting the potential number of studies that could have been included in the systematic review. The confidence in and generalizability of the results are impacted, given that the review is based on a relatively small sample of selected articles. These issues are further compounded by broad heterogeneity among the selected studies in terms of screening tools, patient populations, country of focus, study perspective, and WTP thresholds. In particular, WTP thresholds and study perspectives vary to reflect the structure of the prevailing healthcare systems in each respective country.

The issue of generalizability can be addressed when a more significant number of relevant cost-effectiveness studies on PC screening become available. This should be complemented by standardizing outcome measures, such as cost, QALY, and WTP thresholds, within and, if possible, across countries to facilitate easier comparisons. Prospective studies and those that rely on longitudinal real-world data are also important, and they can be used to validate the costs and benefits of PC screening estimated in economic evaluation studies.

## 5. Conclusions

This study systematically reviewed economic evaluation studies on PC screening and highlighted the main contextual factors that influence cost-effectiveness, namely epidemiological factors, cost, the diagnostic performance of screening tools, and overall study design. Screening was found to be cost-effective compared with no screening among the selected studies, with EUS, MRI, risk-based screening tools, AU, microRNA, and blood-based biomarkers tools being preferred methods to screen individuals at high risk of PC, namely those with a hereditary/familial pathology. One-time and annual screening using EU was generally cost-effective among individuals with familial pancreatic cancer and those with genetic susceptibility syndromes; meanwhile, one-time risk-based screening was generally cost-effective among patients with new-onset diabetes. It is important to note that these results are based on a limited number of included studies in the systematic review, which may be due to the literature search being conducted across a limited number of databases. Notable heterogeneity in study design, target populations, study perspective and country setting should also be considered. Further research is encouraged to support the findings of this review and even to investigate cost-effectiveness in other high-risk population groups and countries not currently covered.

## Data Availability

The protocol for this systematic review is available in the International Prospective Register of Systematic Reviews, PROSPERO, under registration number CRD42023467167: https://www.crd.york.ac.uk/prospero/ (accessed on 14 October 2023). The protocol was registered on 14 October 2023. The search strategy and results of the 28-item CHEERS 2022 checklist for the 31 selected studies are available in the Supplementary Files while the results of the search strategy are included in this manuscript.

## Supplementary Materials

The following supporting information can be downloaded at: https://www.mdpi.com/article/10.3390/curroncol32040225/s1, File S1: Search strategy. Table S1: CHEER checklist. Table S2: Sensitivity and specificity of screening tools. References [25,26,27,28,31,32,33,34,35,36,37] are cited in the Supplementary Materials.

## Abbreviations

The following abbreviations are used in this manuscript:

AU	Abdominal ultrasound
CA 19-9	Carbohydrate antigen 19-9
CAPS	Cancer of the Pancreas Screening Consortium
CT	Computed tomography
CHEERS	Consolidated Health Economic Evaluation Reporting Standards
CE	Cost-effectiveness
END-PAC	Enriching New-Onset Diabetes for Pancreatic Cancer
ERCP	Endoscopic retrograde cholangiopancreatography
EU	Endoscopic ultrasound
FAMMM	Familial atypical mole melanoma
FPC	Familial pancreatic cancer
FNA	Fine needle aspiration
FDR	First-degree relative
HP	Hereditary pancreatitis
ICER	Incremental cost-effectiveness ratio
IPMN	Intraductal papillary mucinous neoplasm
LSD	Long-standing type 2 diabetes
LYS	Life-years saved
MRCP	Magnetic resonance cholangiopancreatography
MRI	Magnetic resonance imaging
NOD	New-onset diabetes
PC	Pancreatic cancer
PDAC	Pancreatic ductal adenocarcinoma
PJS	Peutz–Jeghers syndrome
PRISMA	Preferred Reporting Items for Systematic Reviews and Meta-analyses
PSA	Probabilistic sensitivity analysis
PET	Positron emission tomography
QALY	Quality-adjusted life-years
SEER	Surveillance, Epidemiology, and End Results
THIN	The Health Improvement Network
WTP	Willingness to pay
1-way SA	one-way sensitivity analysis
