# Supplementary material for: A Systematic Review of Cost-Effectiveness Studies on Pancreatic Cancer Screening
Source: Curr Oncol. 2025 Apr 11;32(4):225. doi: 10.3390/curroncol32040225 (PMC12025814; doi:10.3390/curroncol32040225)
Supplement: Supplementary file 1 [file curroncol-32-00225-s001.zip › ESM_1_Search strategy + Screening test performance_1.pdf]

ELECTRONIC SUPPLEMENTARY MATERIALS (ESM)

A systematic review of cost-effectiveness studies on pancreatic cancer screening

Diedron Lewis[1], Laura Jimenez[2], Kelvin K Chan[3], Susan Horton[4], William W L Wong[1]

Corresponding author
Diedron Lewis
d26lewis@uwaterloo.com
ORCID: 0009-0003-4227-7550

[1] School of Pharmacy, University of Waterloo, Waterloo, ON, N2G 1C5, Canada
[2] Department of Community Health and Epidemiology, Dalhousie University, Halifax, NS, B3H 4R2, Canada
[3] Odette Cancer Centre, Sunnybrook Health Sciences Centre, Toronto, ON, M4N 3M5, Canada
[4] School of Public Health Sciences, University of Waterloo, Waterloo, ON, N2L 3G5, Canada

Search strategy

PubMed query

Category	Search terms
#1	(Esophageal Neoplasms [mesh] OR Stomach Neoplasms [mesh] OR Liver Neoplasms [mesh] OR Pancreatic Neoplasms [mesh] OR ((liver[tw] OR esophageal[tw] OR stomach[tw] OR pancreatic[tw]) AND (cancer[tw] OR carcinoma[tw] OR tumor[tw] OR tumour[tw])) OR Liver cancer[tw] OR esophageal cancer[tw] OR oesophageal cancer[tw] OR pancreatic cancer[tw] OR stomach cancer[tw] OR gastric cancer[tw] OR Cirrhosis[tw] OR Hepatocellular[tw] OR Liver Neoplasms[mesh] OR Liver Cirrhosis[mesh] OR Hepatitis C[mesh] OR Hepatoma[tw] OR Hepatoblastoma[tw] OR Tumour[tw] OR adenocarcinoma[tw] OR squamous[tw])
#2	screening[tw] OR Detection[tw] OR Mass screening[mesh] OR Early detection of cancer[mesh])
#3	Cost benefit analysis[mesh] OR Cost analysis[mesh:noexp] OR Economics[subheading] OR Cost effectiveness[ti] OR Cost benefits[ti] OR Cost analysis[ti] OR Economic evaluations[ti] OR Cost utility[ti] OR cost [ti] OR economic*[ti])
#1 AND #2 AND #3	(Esophageal Neoplasms [mesh] OR Stomach Neoplasms [mesh] OR Liver Neoplasms [mesh] OR Pancreatic Neoplasms [mesh] OR ((liver[tw] OR esophageal[tw] OR stomach[tw] OR pancreatic[tw]) AND (cancer[tw] OR carcinoma[tw] OR tumor[tw] OR tumour[tw])) OR Liver cancer[tw] OR esophageal cancer[tw] OR oesophageal cancer[tw] OR pancreatic cancer[tw] OR stomach cancer[tw] OR gastric cancer[tw] OR Cirrhosis[tw] OR Hepatocellular[tw] OR Liver Neoplasms[mesh] OR Liver Cirrhosis[mesh] OR Hepatitis C[mesh] OR Hepatoma[tw] OR Hepatoblastoma[tw] OR Tumour[tw] OR adenocarcinoma[tw] OR squamous[tw]) AND (screening[tw] OR Detection[tw] OR Mass screening[mesh] OR Early detection of cancer[mesh]) AND (Cost benefit analysis[mesh] OR Cost analysis[mesh:noexp] OR Economics[subheading] OR Cost effectiveness[ti] OR Cost benefits[ti] OR Cost analysis[ti] OR Economic evaluations[ti] OR Cost utility[ti] OR cost [ti] OR economic*[ti])

#5	Search: #2 AND #3 AND #4
#4	Search: (Cost benefit analysis[mesh] OR Cost analysis[mesh:noexp] OR Economics[subheading] OR Cost effectiveness[ti] OR Cost benefits[ti] OR Cost analysis[ti] OR Economic evaluations[ti] OR Cost utility[ti] OR cost [ti] OR economic*[ti])
#3	Search: screening[tw] OR Detection[tw] OR Mass screening[mesh] OR Early detection of cancer[mesh]

#2	Search: (Esophageal Neoplasms [mesh] OR Stomach Neoplasms [mesh] OR Liver Neoplasms [mesh] OR Pancreatic Neoplasms [mesh] OR ((liver[tw] OR esophageal[tw] OR stomach[tw] OR pancreatic[tw]) AND (cancer[tw] OR carcinoma[tw] OR tumor[tw] OR tumour[tw])) OR Liver cancer[tw] OR esophageal cancer[tw] OR oesophageal cancer[tw] OR pancreatic cancer[tw] OR stomach cancer[tw] OR gastric cancer[tw] OR Cirrhosis[tw] OR Hepatocellular[tw] OR Liver Neoplasms[mesh] OR Liver Cirrhosis[mesh] OR Hepatitis C[mesh] OR Hepatoma[tw] OR Hepatoblastoma[tw] OR Tumour[tw] OR adenocarcinoma[tw] OR squamous[tw])

Scopus query

Category	Search terms
#1	((TITLE-ABS-KEY ("Esophageal Neoplasms" OR "Stomach Neoplasms" OR "Liver Neoplasms" OR "Pancreatic Neoplasms") OR TITLE-ABS-KEY (("liver" OR "esophageal" OR "Hepatocellular" OR "gastric" OR "stomach" OR "pancreatic" OR "Hepatoma" OR "Hepatitis C" OR "Cirrhosis") W/3 ("cancer" OR "carcinoma" OR "tumor" OR "tumour" OR "adenocarcinoma" OR "squamous")) OR TITLE-ABS-KEY ("Hepatoblastoma")))
#2	(TITLE-ABS-KEY ("screening" OR "Detection" OR "Mass screening" OR "Early detection of cancer"))
#3	(TITLE-ABS-KEY ("Cost benefit*" OR "Cost analysis" OR "Cost effectiveness" OR "Economic evaluations" OR "Cost utility"))
#1 and #2 and #3	((TITLE-ABS-KEY ("Esophageal Neoplasms" OR "Stomach Neoplasms" OR "Liver Neoplasms" OR "Pancreatic Neoplasms") OR TITLE-ABS-KEY (("liver" OR "esophageal" OR "Hepatocellular" OR "gastric" OR "stomach" OR "pancreatic" OR "Hepatoma" OR "Hepatitis C" OR "Cirrhosis") W/3 ("cancer" OR "carcinoma" OR "tumor" OR "tumour" OR "adenocarcinoma" OR "squamous")) OR TITLE-ABS-KEY ("Hepatoblastoma"))) AND (TITLE-ABS-KEY ("screening" OR "Detection" OR "Mass screening" OR "Early detection of cancer")) AND (TITLE-ABS-KEY ("Cost benefit*" OR "Cost analysis" OR "Cost effectiveness" OR "Economic evaluations" OR "Cost utility"))

Table S1: Sensitivity and specificity of screening tools

**References**	Test sensitivity	Test specificity
Wang et al., 2022 [1]	N/A	N/A
Schwartz et al., 2022 [2]	Risk-based screening tool: 78%	Risk-based screening tool: 92%
Rulyak et al., 2003 [3]	EUS: 90%	N/A
Peters et al., 2024 [4]	MRI with EUS ≥ 62%	96%
Kumar et al., 2021 [5]	EUS: 71.25%	EUS: 99.82%
Kowada 2022 [6]	MicroRNA: 98%<br>CA 19-9: 79%<br>AU: 88%<br>MRI: 93%<br>EUS: 91%<br>CT: 90%<br>PET: 89%	MicroRNA: 85%<br>CA 19-9: 82%<br>AU: 94%<br>MRI: 89%<br>EUS: 86%<br>CT: 87%<br>PET: 70%
Kowada 2020 [7]	AU: 88%<br>CT: 90%<br>EUS: 91%<br>MRI: 93%<br>PET: 89%	AU: 94%<br>CT: 87%<br>EUS: 86%<br>MRI: 89%<br>PET: 70%
Joergensen et al., 2016 [8]	N/A	N/A
Draus et al., 2023 [9]	Biomarker test: 80-99%	Biomarker test: 80-99%
Corral et al., 2019 [10]	EUS: 90%<br>MRI: 80%	EUS: 90%<br>MRI: 80%

AU - abdominal ultrasound; CT - computed tomography; MRI - magnetic resonance imaging; PET - positron emission tomography; CA 19-9 - carbohydrate antigen 19-19; EUS -endoscopic ultrasound; N/A – not available

References

[1] Wang L, Scott FI, Boursi B, Reiss KA, Williams S, Glick H, et al. Cost-effectiveness of a risk-tailored pancreatic cancer early detection strategy among patients with new-onset diabetes. Clinical Gastroenterology and Hepatology 2022;20:1997-2004.e7. https://doi.org/10.1016/j.cgh.2021.10.037.

[2] Schwartz NRM, Matrisian LM, Shrader EE, Feng Z, Chari S, Roth JA. Potential cost-effectiveness of risk-based pancreatic cancer screening in patients with new-onset diabetes. Journal of the National Comprehensive Cancer Network 2022;20:451–9. https://doi.org/10.6004/jnccn.2020.7798.

[3] Rulyak SJ, Kimmey MB, Veenstra DL, Brentnall TA. Cost-effectiveness of pancreatic cancer screening in familial pancreatic cancer kindreds. Gastrointest Endosc 2003;57:23–9. https://doi.org/10.1067/mge.2003.28.

[4] Peters MLB, Eckel A, Seguin CL, Davidi B, Howard DH, Knudsen AB, et al. Cost-effectiveness analysis of screening for pancreatic cancer among high-risk populations. JCO Oncol Pract 2024;20:278–90. https://doi.org/10.1200/OP.23.00495.

[5] Kumar S, Saumoy M, Oh A, Schneider Y, Brand RE, Chak A, et al. Threshold analysis of the cost-effectiveness of endoscopic ultrasound in patients at high risk for pancreatic ductal adenocarcinoma. Pancreas 2021;50:807–14. https://doi.org/10.1097/MPA.0000000000001835.

[6] Kowada A. Cost-effectiveness of MicroRNA for pancreatic cancer screening in patients with diabetes. Pancreas 2022;51:1019–28. https://doi.org/10.1097/MPA.0000000000002130.

[7] Kowada A. Cost-effectiveness of abdominal ultrasound versus magnetic resonance imaging for pancreatic cancer screening in familial high-risk individuals in Japan. Pancreas 2020;49:1052–6. https://doi.org/10.1097/MPA.0000000000001614.

[8] Joergensen MT, Gerdes A-M, Sorensen J, Schaffalitzky de Muckadell O, Mortensen MB. Is screening for pancreatic cancer in high-risk groups cost-effective? – Experience from a Danish national screening program. Pancreatology 2016;16:584–92. https://doi.org/10.1016/j.pan.2016.03.013.

[9] Draus T, Ansari D, Andersson R. Model-based screening for pancreatic cancer in Sweden. Scand J Gastroenterol 2023;58:534–41. https://doi.org/10.1080/00365521.2022.2142481.

[10] Corral JE, Das A, Bruno MJ, Wallace MB. Cost-effectiveness of pancreatic cancer surveillance in high-risk individuals. Pancreas 2019;48:526–36. https://doi.org/10.1097/MPA.0000000000001268.
